# Low and Moderate Doses of Caffeinated Coffee Improve Repeated Sprint Performance in Female Team Sport Athletes

**DOI:** 10.3390/biology11101498

**Published:** 2022-10-13

**Authors:** Raci Karayigit, Scott C. Forbes, Zakir Osmanov, Canan Yilmaz, Burak Caglar Yasli, Alireza Naderi, Hakan Buyukcelebi, Daniela Benesova, Tomasz Gabrys, Ozcan Esen

**Affiliations:** 1 Faculty of Sport Sciences, Ankara University, Gölbaşı, Ankara 06830, Turkey; 2 Department of Physical Education Studies, Brandon University, Brandon, MB R7A 6A9, Canada; 3 Department of Medical Biochemistry, Faculty of Medicine, Gazi University, Ankara 06500, Turkey; 4 Department of Coaching Education, Faculty of Sport Sciences, Iğdır University, Iğdır 76000, Turkey; 5 Department of Sport Physiology, Boroujerd Branch, Islamic Azad University, Boroujerd 6915136111, Iran; 6 Department of Coaching Education, Faculty of Sport Sciences, İnönü University, Malatya 44000, Turkey; 7 Department of Physical Education and Sport, Faculty of Education, University of West Bohemia, 30100 Pilsen, Czech Republic; 8 Department of Sport, Exercise and Rehabilitation, Northumbria University, Newcastle-upon-Tyne NE1 8ST, UK

**Keywords:** ergogenicity, exercise performance, catecholamines, tolerance, anaerobic exercise

## Abstract

**Simple Summary:**

The bulk of research on caffeine as an ergogenic aid has been on endurance performance, however there is evidence that caffeine can enhance short-term high-intensity performance. Caffeine may have an ergogenic impact during anaerobic exercise by facilitating central effects by antagonizing adenosine receptors, hence decreasing the detrimental effects of adenosine on neurotransmission, arousal, and pain perception. Caffeine intake also activates the central nervous system, which promotes alertness and concentration. Additionally, athletes typically consume coffee containing caffeine. To date, the bulk of study has focused on the administration of 3–9 mg/kg of anhydrous caffeine, as opposed to the readily accessible source of caffeine, coffee, and has mostly been conducted on males. Consequently, there is a dearth of study evaluating the ergogenic impact of caffeine on females, particularly when consuming alternative caffeine delivery methods, such as coffee. The current study is the first to investigate the effects of low (3 mg/kg) and moderate (6 mg/kg) doses of caffeine from coffee on repeated sprint performance in females.

**Abstract:**

The aim of this study was to determine the effect of low and moderate doses of caffeine ingestion via caffeinated coffee on repeated sprint test (RST) and plasma catecholamine concentration in trained female team-sport athletes. In a randomized, double-blind, crossover design, 13 female team-sport athletes (VO_2max_: 48.7 ± 4 mL·kg·min^−1^) completed three RST trials, separated by 4-day, 60 min post-ingestion of either 3 mg·kg^−1^ (LCOF) or 6 mg·kg^−1^ (MCOF) or placebo (PLA). The RST consisted of 12 × 4 s sprints on a cycle ergometer interspersed with 20 s of active recovery. Blood lactate (BLa) and glucose (GLU) and epinephrine and norepinephrine concentrations were collected before and 60 min after coffee ingestion, and after RST. Heart rate (HR) and ratings of perceived exertion (RPE) were measured at the beginning of RST, and after the 6th and 12th sprints. Average peak power score during RST was significantly improved after LCOF (*p* = 0.016) and MCOF (*p* = 0.041) compared to PLA, but peak and mean power output of the individual sprints, and fatigue index were not different between trials (all *p* > 0.05). Epinephrine and norepinephrine concentrations were significantly higher before and after RST in LCOF and MCOF compared to PLA (all *p* < 0.05). BLa was also higher after RST in both LCOF and MCOF compared to PLA (*p* = 0.005). HR, RPE, and GLU were not different between conditions (*p* > 0.05). In conclusion, low and moderate dose of caffeine ingestion can enhance the average peak power score during repeated sprints. These findings partly support low and moderate doses of caffeine supplementation via coffee as a nutritional ergogenic aid for trained female team-sport players during repeated sprint exercise.

## 1. Introduction

Caffeine is one of the most popular ergogenic aids in sport and the plethora of research has investigated the physiologic, metabolic, and performance effects of caffeine in the last decades [1]. Regarding the mechanism of action, caffeine exerts its ergogenic benefits via the facilitation of central effects by antagonising adenosine receptors, thereby increasing neurotransmission [2], motor-unit recruitment [3], and release of neurotransmitters such as dopamine and catecholamine [4,5]. However, given that enhanced physical performance was reported during endurance exercise without an increase in blood epinephrine (Ep) concentration following caffeine supplementation [6], the increase of catecholamine concentrations appears to have a lesser impact [7]. However, there is lack of studies assessing if there could be an association between the elevation of catecholamine concentrations and repeated sprint performance; therefore, further research is needed. Caffeine supplementation has been reported to have an ergogenic effect on muscle endurance and strength, anaerobic power, and aerobic endurance, which are crucial performance parameters for team-sports players [1]. Repeated sprints and power, which are commonly measured via Wingate test, are also critical variables regarding team-sport performance, but the literature reported inconsistent findings about the effect of caffeine supplementation on these variables [8,9,10]. For example, caffeine supplementation has been reported to improve mean power output (MPO) and sprint work during 18 × 4 s sprints with 2 min active recovery in amateur male team-sport players [11]. A subsequent study reported that caffeine supplementation improved sprint performance (e.g., MPO and fatigue index) with a 90 s rest interval, but not with a 20 s interval in recreational male athletes [12]. Therefore, further studies are needed to evaluate the effect of caffeine supplementation on repeated sprints and power, which would improve understanding of the potential ergogenity of caffeine supplementation regarding team-sport performance.

Since caffeine has been reported to enhance physical performance in both male [13] and female athletes’ [14,15], and since recent evidence suggests that the ergogenic effect of caffeine is of similar magnitude between sexes [16,17], current guidelines for caffeine supplementation are similarly implemented for both sexes. However, it is important to note that these guidelines were established primarily from studies developed in males, and females are heavily underrepresented in this field of research. Further, no study, to our knowledge, has assessed repeated sprint performance in exclusively female team-sport players following both low and moderate dose of caffeine supplementation.

The previous studies outlined above, which investigated the effect of caffeine supplementation on repeated sprints, administered moderate dose of caffeine (5 and 6 mg·kg^−1^, respectively) [8,11]. Although these doses are in the current recommended range, the ergogenic effect of low doses of caffeine (3 mg·kg^−1^) has been also considered and/or reported in team-sport athletes [13,18]. However, there is a lack of studies comparing the effect of low and moderate doses of caffeine supplementation in both repeated sprints and team-sports players, which are areas requiring further studies. There are various forms in which to ingest caffeine prior to exercise, including anhydrous caffeine, energy drinks, chewing gum, bars, jelly, aerosols, and coffee [19]. Coffee, which includes other ingredients in addition to caffeine, was previously proposed to attenuate the ergogenic benefits of caffeine compared to ingestion of the same dose in the form of anhydrous caffeine [16]. However, more recent investigations have reported that 5 mg·kg^−1^ of caffeine in the form of coffee or anhydrous caffeine provided similar ergogenic effects on endurance [20] and resistance exercise [21]. Interestingly, Clarke et al. [22] reported that 3 mg·kg^−1^ of caffeine ingestion either in the form of anhydrous caffeine or coffee did not enhance repeated sprint performance in untrained males. The lack of effect in the study of Clarke et al. might be due to the administration of low dose or training status of participants. However, it is more likely to suggest that the dose of caffeine may modulate the ergogenic effects of caffeine, given it has been recently reported that trained and untrained individuals experience similar improvements in performance following caffeine ingestion [23]. However, habitually, the strategy adopted by most athletes is to use a low dose of caffeine to reduce the prevalence and magnitude of any potentially negative side-effects while achieving performance enhancing results [24]. Therefore, the aim of this study was to determine the effects of a low (3 mg·kg^−1^) and a moderate dose of caffeine (6 mg·kg^−1^) through caffeinated coffee on repeated sprint test (RST) and plasma catecholamine concentrations in trained female team-sport athletes. The hypotheses of the study were that both doses of caffeine would enhance RST performance.

## 2. Materials and Methods

### 2.1. Participants

Thirteen (mean ± SD, age: 20.0 ± 1 years, body mass: 61.9 ± 7.4 kg, height 167.2 ± 5.7 cm, VO_2max_ 48.7 ± 4 mL·kg·min^−1^) trained, healthy, non-smoker team-sport (i.e., soccer, basketball, and handball) female players participated in this study. All participants had at least 5 years’ experience competing in national league. All participants were involved in a regular training program, with an average of 5 ± 1 days team-sport based training sessions a week. Participants did not currently, or in the previous 3 months, use ergogenic aids (e.g., creatine, beta-alanine, nitrate), except caffeine, that could affect muscular performance. Daily caffeine intake was determined using an adapted version of a questionnaire proposed by Buhler et al. [25]. The questionnaire was administered under the supervision of a qualified nutritionist. Furthermore, the amount of caffeine ingested from food/drink sources were used to assess the total daily caffeine intake. Based on the assessment, all participants were considered habitual caffeine consumers (5.4 mg·kg^−1^) according to Filip et al. [26]. All participants declared having regular menstrual cycles with no more than 3 days variation in the range of their menstrual cycles’ length for the previous 4 months tracked via a mobile application (Menstruation Calendar, Period Track, Istanbul, Turkey) [14]. Participants were informed about the risks associated with the study and signed a written consent form before initiating the experimental protocols. The study was conducted in accordance with the Declaration of Helsinki (2008) and approved by the local University Clinical Research Ethic Committee (16-1015-17).

### 2.2. Experimental Protocol

**Figure 1:**
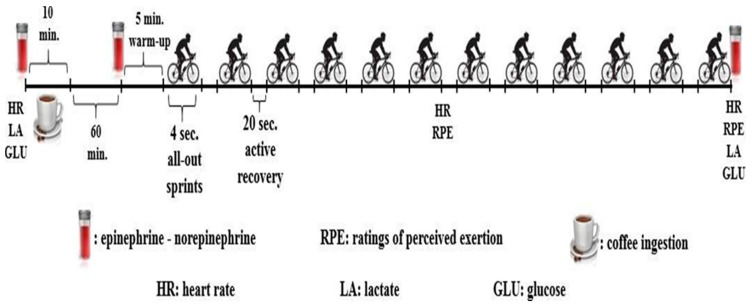
Schematic representation of the repeated sprint test protocol.

Participants completed five separate visits over 15 days. On the first visit, all participants performed an incremental cycling protocol to exhaustion, as described elsewhere [27], to determine VO_2max_. On the second visit, participants were familiarized with the RST on a Monark cycle ergometer (Ergomedic 894E, Vansbro, Sweden), and to using the Borg’s 6–20 rating of perceived exertion (RPE) scale [28]. Following completion of this familiarization session, participants were assigned to consume either a low dose (LCOF) or moderate dose of caffeinated coffee (MCOF) or decaffeinated coffee as a placebo (PLA), in a randomized, double-blind, cross-over design. A period of 96 h separated the LCOF, MCOF, and PLA. Venous blood samples were collected before and 60 min after coffee ingestion, and after RST for blood ephedrine and norephedrine concentration. Capillary blood lactate (BLa, Lactate Plus, Nova Biomedical, NY, USA) and glucose concentrations (GLU, Accutrend Plus, Roche, Berlin, Germany) were measured before and after RST. Participants’ heart rate (HR) (Polar Team2, Espoo, Finland) and RPE were also recorded before coffee ingestion (only HR), and at 6th and 12th sprint. The experimental trials were all carried out at the similar time of day (between 08.00–11.00 a.m.) after a 10 h overnight fast. Participants were asked to refrain from vigorous exercise in the 48 h, and caffeine ingestion in the 24 h, prior to testing. Participants recorded their 24 h dietary intake before the first experimental trial and replicated same diet in the 24 h before subsequent trials. Prior to the experimental sessions, a comprehensive list of foods and medicines containing caffeine was also provided to participants. Although caffeine increases sprint cycling performance with a similar magnitude in all three phases of the menstrual cycle [14], test sessions were performed during the luteal phase which was determined by individual declaration of the 20th day from the beginning of menses [14]. Figure 1 displayed a schematic of the experimental protocol.

### 2.3. Repeated Sprint Test

After setting up individual seat and handle positions, which were recorded and replicated in the subsequent sessions for each participant, participants began a standardized 5 min warm-up against a load of 1 kg. Following the warm-up, participants performed 12 × 4 s “all-out” sprints interspersed with 20 s of active recovery [12]. A flywheel braking force corresponding to 7.5% of the participant’s body mass was applied during the sprints. The test was automatically initiated by Monark test software when the participant reached ≥110 rpm during unloaded pedalling and subsequent instant application of the load. Participants were asked to remain seated for the entire duration of each sprint to prevent the recruitment of other muscle groups. All participants were given strong verbal encouragement to cycle maximally and pedal as fast as possible for each 4 s sprint. After each sprint, participants pedalled at 60 rpm against no load for the 20 s active recovery phases [12]. Peak (PPO) and mean power output (MPO) were calculated via Monark Software (Version 3.3.0.0, Vansbro, Sweden). Fatigue index (FI, %) was calculated via the formula proposed by Glaister et al. [29].

### 2.4. Supplementation Protocol Coffee Preparation

Participants consumed 3 Nescafe Gold (Nestle, İstanbul, Turkey) instant coffees with caffeinated and decaffeinated versions used and dissolved in 500 mL of hot water (approximately 65 °C) and served in a big glass cup. Participants were given 10 min to ingest the beverage, followed by 60 min of passive rest. All trials used the same coffee brand, which contained 36 mg of caffeine per 1 gr of coffee [30]. Participants ingested 0.16 g·kg^−1^ of caffeinated coffee for MCOF to achieve the consumption of 6 mg·kg^−1^ of caffeine or 0.16 g·kg^−1^ of decaffeinated coffee for PLA to produce ~0 mg·kg^−1^ of caffeine or 0.08 g·kg^−1^ caffeinated + 0.08 g·kg^−1^ decaffeinated coffee (totally 0.16 g·kg^−1^) for LCOF to achieve an intake of 3 mg·kg^−1^ of caffeine.

### 2.5. Blood Sampling

Venous blood samples (5 mL) were collected into a serum separator tube before consuming coffee and one hour after completing coffee intake, and, immediately post-exercise, were centrifuged at 4000 rpm for 10 min. Serum was subsequently removed and stored at −80 °C for the later analysis of both serum epinephrine and norepinephrine. Serum epinephrine and norepinephrine concentrations were analysed by using commercial Sandwich-ELISA kits (catalogue number for epinephrine: 201-12-1039, and for norepinephrine: 201-12-0987) (Sunred, Beijing, China). Both intra- and inter-batch coefficient of variations were <10%. Results are presented as pg/mL.

### 2.6. Statistical Analyses

All variables presented a normal distribution according to the Shapiro–Wilk test. A two-way (supplement × time) repeated-measures ANOVA was used to assess differences in PPO, MPO, HR, GLU, BLa, RPE, and catecholamine. Differences in FI and average of peak and mean power for the 12 sprints performed during the RST were analysed with a one-way ANOVA for repeated measures. Effect sizes were calculated as partial eta squared (η_p_^2^), defined as trivial (<0.10), moderate (0.25–0.39), or large (≥0.40) [31]. Where the ANOVA shows a significant effect, paired samples *t*-tests were employed using Bonferroni corrected paired *t*-tests to define the origin of any potential effect. Statistical significance was set at *p* < 0.05, and all data were analysed using SPSS 23.0 (IBM Corp., Armonk, NY, USA), and are presented as mean ± SD. Effect size was expressed as 95% confidence interval (CI).

## 3. Results

### 3.1. Repeated Sprint Performance

**Figure 2:**
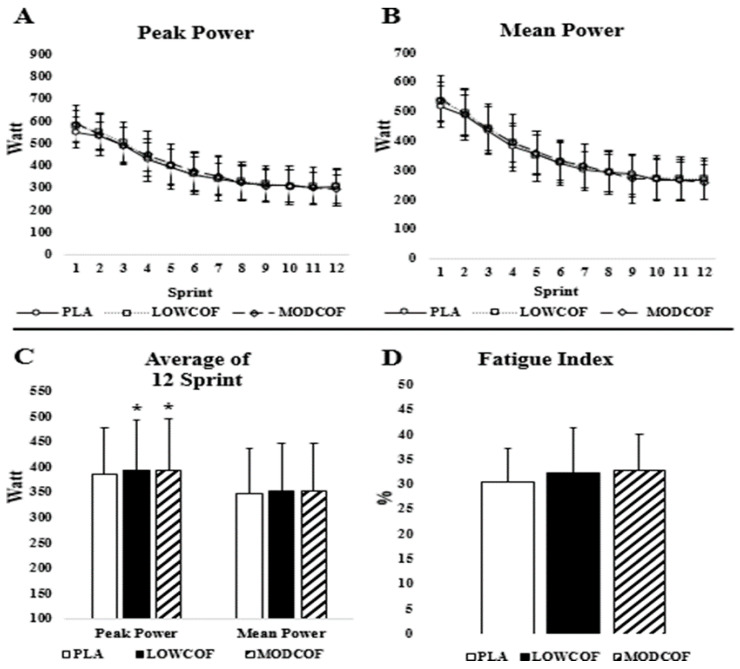
Peak power (**A**) and mean power (**B**) for each sprint across intervals, average peak and mean power output of the 12 × 4 s repeated sprints (**C**), and fatigue index during 12 × 4 s repeated sprints (**D**) test with ingestion of placebo (PLA) or 3 mg·kg^−1^ of caffeine from coffee (LCOF) or 6 mg·kg^−1^ of caffeine from coffee (MCOF). *: Significantly different from PLA at *p* < 0.05.

There was no condition × sprint interaction for PPO (F = 1.036, *p* = 0.420, η_p_^2^ = 0.079) and MPO (F = 0.870, *p* = 0.646, η_p_^2^ = 0.068) (Figure 2A,B). There was no significant main effect for condition in PPO (F = 0.558, *p* = 0.580, η_p_^2^ = 0.044) and MPO (F = 0.265, *p* = 0.770, η_p_^2^ = 0.022). Although there was no interaction effect, 12 of 13 participants responded to LCOF in the first sprint and increased PPO by an average of 4.7% compared to PLA. Similarly, 12 of 13 participants improved PPO by an average of 6.5% after the ingestion of MCOF compared to PLA. Low and moderate doses of caffeine significantly increased average peak power score of 12 total sprints (F = 4.586, *p* = 0.022, η_p_^2^ = 0.294). Post-hoc analysis revealed that LCOF (402.74 ± 86 W, *p* = 0.016, 95% CI = 1.6–13.0) and MCOF (404.22 ± 76.79 W, *p* = 0.041, 95% CI = 0.3–16.0) significantly enhanced average peak power score compared to PLA (386.95 ± 72.07, Figure 2C), but there was no difference between LCOF and MCOF (*p* = 0.768). Caffeine conditions did not enhance average mean power score of 12 total sprints (F = 2.885, *p* = 0.077, η_p_^2^ = 0.208). There was also no significant difference in FI between conditions (F = 0.868, *p* = 0.432, η_p_^2^ = 0.067) (Figure 2D).

### 3.2. Catecholamines

**Figure 3:**
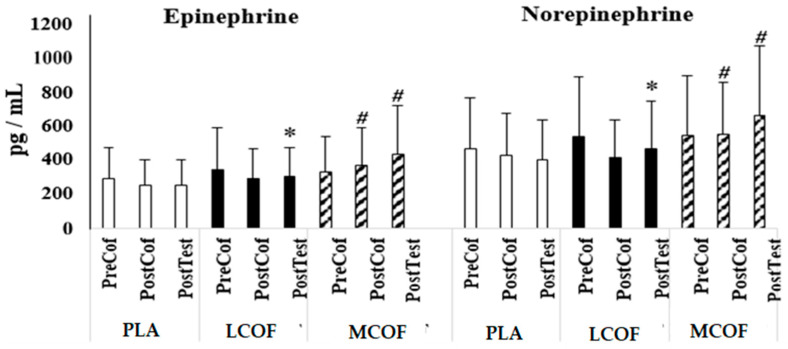
Blood epinephrine and norepinephrine concentrations measured at PreCof (Prior to coffee ingestion), PostCof (60 min after coffee ingestion), and Posttest (immediately after test protocol) with the ingestion of placebo (PLA) or 3 mg·kg^−1^ of caffeine from coffee (LCOF) or 6 mg·kg^−1^ of caffeine from coffee (MCOF). *: Significantly different from PLA; #: Significantly different from PLA and LCOF.

Low and moderate doses of caffeine intake significantly increased epinephrine (F = 9.749, *p* = 0.001, η_p_^2^ = 0.520). Before coffee ingestion, epinephrine concentration was not different between conditions (*p* > 0.05), while it was higher in MCOF compared to LCOF (*p* = 0.006, 95% CI = 28.3–129.4) and PLA (*p* = 0.004, 95% CI = 47.4–183.9) 60 min after coffee ingestion. Immediately after the RST, epinephrine concentration was also higher in LCOF (*p* = 0.001, 95% CI = 25.2–68.5) and MCOF (*p* = 0.005, 95% CI = 66.7–287.2) compared to PLA. MCOF also resulted in higher epinephrine concentration than LCOF (*p* = 0.017, 95% CI = 29.3–230.8). There was a significant main effect for norephedrine concentration following the caffeine conditions (F = 6.214, *p* = 0.009, η_p_^2^ = 0.408). Similar to epinephrine, before coffee ingestion, norepinephrine concentration was not different between conditions (*p* > 0.05), while it was significantly higher in MCOF compared to LCOF (*p* = 0.046, 95% CI = 3.3–266.0) but not to PLA (*p* = 0.058) 60 min after coffee ingestion. There was also no difference between LCOF and PLA (*p* = 0.678). Immediately after RST, norepinephrine concentration was significantly higher in LCOF (*p* = 0.027, 95% CI = 9.3–123.0) and MCOF (*p* = 0.004, 95% CI = 107.7–420.4) compared to PLA. MCOF also resulted in higher norephedrine concentration than LCOF (*p* = 0.007, 95% CI = 68.0 –327.7) (Figure 3).

### 3.3. Heart Rate, RPE, Glucose, and Lactate

HR increased over time (*p* = 0.001) but was not different between conditions (F = 0.774, *p* = 0.472, η_p_^2^ = 0.061). Similarly, RPE increased from 6th to 12th sprint (*p* = 0.001) with no differences between conditions (F = 0.149, *p* = 0.862, η_p_^2^ = 0.012). There was a significant time effect (*p* = 0.001) for glucose with no significance differences between conditions (F = 1.473, *p* = 0.249, η_p_^2^ = 0.109). However, BLa was significantly different between conditions (F = 9.597, *p* = 0.001, η_p_^2^ = 0.444). Post-hoc analysis showed LCOF (*p* = 0.007, 95% CI = 0.2–1.5) and MCOF (*p* = 0.005, 95% CI = 0.3–1.6) were significantly higher compared to PLA, as shown in Table 1.

## 4. Discussion

This study evaluated the effects of a low (3 mg·kg^−1^) and a moderate dose of caffeine (6 mg·kg^−1^) ingestion via coffee on repeated sprint performance and catecholamine response in trained female team-sport athletes. The main finding was that low and moderate doses of coffee ingestion improved the average PPO attained by participants during the repeated sprint test compared to placebo, with no differences between doses (3 mg·kg^−1^ vs. 6 mg·kg^−1^). Furthermore, ephedrine and norephedrine concentrations were higher before and after RST following the low and moderate doses of caffeine ingestion compared to placebo. In addition, a distinct effect was observed between the caffeine doses. However, PPO and MPO of the individual sprints, and FI were similar between LCOF, MCOF, and PLA. The findings of this study support, at least partly, the results of several previous studies that reported ergogenity of caffeine for repeated sprint performance. In practical terms, low or moderate doses of caffeine ingestion in the form of coffee may be of value to female team-sport athletes seeking to improve repeated sprint performance, at least on average.

Greater average PPO during RST in the present study supports previous observations reporting enhanced repeated sprint exercise after caffeine ingestion in trained males [4,32] and females [15,33]. More specifically, improved average PPO during RST is consistent with a previous study reporting enhanced average PPO during two sets of 18 × 4 s all-out sprints in well-trained male team-sport players following 6 mg·kg^−1^ of caffeine ingestion [11]. Our finding extends the findings of this previous study and suggests that a similar performance benefit can be gained by trained female team-sports players. Additionally, our findings reveal that female team-sport athletes can gain the same benefits from a low dose of caffeine (~3 mg·kg^−1^) as much as from a higher dose (~6 mg·kg^−1^) Thus, a dose of ~3 mg·kg^−1^ caffeine might be suggested as a ceiling effect for female team-sport athletes and sprint performance. Further, it can be assumed that with the lower doses, female athletes can see similar benefits without the risk of side effects caused by high doses. However, our findings conflict with another previous study that found no benefits of caffeine ingestion on repeated sprint performance (2 × 60 s) in recreational individuals (five females) [34]. Besides inter-study differences (e.g., participant training status and sex differences), the most obvious explanation for the discrepant findings between the present and previous study is the exercise protocol differences; the previous study applied a considerably longer sprint duration and lesser number of sprints. In team sports, however, athletes are required to perform frequent repetitive all-out sprints with short recovery periods to overcome opponents. Therefore, the results of the present study support the use of caffeinated coffee to be ergogenic for repeated sprint exercise, at least for improving average PPO, in female team-sport athletes.

With regard to individual sprints, PPO, MPO, and FI did not differ following ingestion of neither a low nor moderate dose of caffeine. Consistent with these findings, Lee et al. [12] reported that caffeine ingestion did not enhance individual sprint performance with a 20 s rest interval, but improved with a 90 s interval, suggesting the ergogenity of caffeine might depending on rest interval between sprints. However, although not significant in the univariate analyses, PPO was improved in the first sprint by 4.7% and 6.5% following low and moderate doses of caffeine ingestion compared to placebo, respectively, in 12 of the 13 participants in the present study. It can be suggested that caffeine may benefit performance during initial effort, but its ergogenity disappears as fatigue develops, likely due to an increase in the by-products of anaerobic metabolism [11,12]. Supporting this, the results of the present study, in accordance with previous studies [11,12], revealed high BLa after RST in the caffeine conditions, which may be attributed to an increase in anaerobic work performed during the first sprints. Having higher blood lactate without differences in subjective fatigue can be considered meaningful. Participants were able to produce more power, at least in the initial stages, which resulted in larger metabolic by-product accumulation, but the greater accumulation of BLa does not change perception of effort, likely due to increased neural drive. This can be considered as a practical application for team sports where cognition and attention decrease with increased fatiguing work. It has been also suggested that caffeine may inhibit blood lactate clearance rather than production [16], since the 20 s rest between sprints in the present study was not enough for the liver to metabolize lactate or obstruct muscular glycogenolysis [35,36]. Overall, the 4.7–6.5% improvement during the initial sprint is important, given that it could be translated to individual sports, such as track, where one sprint is all an athlete will perform, and that track can even be a team sport if you consider short distance relays.

In the present study, both 3 and 6 mg·kg^−1^ of caffeine resulted in similar benefits in RST, which is line with many [13,31,37], but not all [9], previous studies. In particular, and similar to our results, 3 mg·kg^−1^ of caffeine was reported to improve PPO during a 15 s cycling sprint [17], peak speed during 7 × 30 m running sprints [33], and running distance covered at >18 km/h during a real game in female athletes [15]. However, coffee providing 3 mg·kg^−1^ of caffeine was suggested to have little effect on repeated sprint cycling performance in relatively untrained males [22]. Additionally, it has been reported that 280 mg of caffeine from coffee had no effect on PPO during a 30s Wingate test [38]. A more recent study, however, reported that both coffee and caffeine anhydrous had an ergogenic effect on the repeated sprint test, with no clear difference between the two forms. Combined with the observations of this previous study, findings of the present study suggest that coffee providing 3 mg/kg of caffeine may be considered a suitable source of caffeine supplementation before repeated sprint exercise.

In addition to the dose and form, participants’ level of habituation to caffeine may also modify the ergogenic effect of caffeine [39]. Durkalec-Michalski et al. [40] suggested that a higher dose of caffeine (6–9 mg·kg^−1^) than current recommendation (3–6 mg·kg^−1^) would be needed to enhance exercise performance in judo athletes habituated to caffeine. Additionally, Beaumont et al. [41] and Lara et al. [42] reported that 3–4 weeks of 3 mg·kg^−1^ of caffeine consumption develops tolerance on endurance exercise performance, while the frequency of some side effects associated to caffeine intake may increase over time [43]. In contrast to these results, the current study showed that both 3 and 6 mg·kg^−1^ of caffeine from coffee enhanced PPO during RST in habitual caffeine (5.4 mg·kg^−1^) consumers. Interestingly, Pickering and Kiely [39] speculated that individuals with high level caffeine usage need to ingest acute caffeine doses greater than their habitually using level to overcome habituation. This is not supported by the current study, because both doses below and above their habitual caffeine intake were effective at producing some ergogenic benefit. Moreover, 200 mg of caffeine was also found to attenuate the sprint performance decrement during RST in low (<40 mg/day) but not moderate-to-high (>130 mg/day) habitual caffeine user males [13]. Although more research is warranted to determine whether habituation impacts exercise performance in female athletes, it seems that habituated athletes still can obtain benefits from low and moderate doses of caffeine. Further investigations should determine whether dishabituation to caffeine in these individuals may increase the ergogenic benefits of acute caffeine intake.

Caffeine ingestion caused a significant elevation of epinephrine and norepinephrine concentrations during the RST compared to the placebo, which is consistent with previous findings [5,7]. The increase in catecholamine concentration after caffeine ingestion may suggest a greater stimulation of the central nervous system [7]. Antagonist of adenosine receptors and/or stimulation of adrenal gland may be the mechanisms of caffeine’s stimulatory effect on catecholamine [7]. However, the observed performance effects in the present study cannot be just attributed to increased catecholamine concentration, since this mechanism has been dismissed by some other studies [44,45,46]. Further, there are several other possible mechanisms, including increased sodium–potassium ATPase activity [19] and mobilization of intracellular calcium [39], that might explain improved average PPO during RST in the present study. Although those mechanisms have been investigated during varying intensities and modalities of exercise [3,4,9,16], these exercises were mostly endurance based. Therefore, further studies are needed to determine the exact mechanism(s) responsible for the ergogenic effects of caffeine on repeated sprint exercise.

The present study has several limitations. To standardise macronutrient intake prior to experimental trials, a 24 h diet record was used and replicated, which may be considered as an acceptable method, however, macronutrient intake prior to each trial was not analysed by a software. Future studies might control pre-test diet more rigorously or/and provide a standardised pre-test diet. Further, although our RST simulates team sport movement patterns [47], current results need to be confirmed in field-based tests and other more ecologically valid scenarios.

## 5. Conclusions

The results of the present study showed that both 3 and 6 mg·kg^−1^ doses of caffeine ingestion increased plasma catecholamine concentration and improved the average peak power score during a RST, but not individual sprint, in trained female team-sport players. These findings partly support low and moderate doses of caffeine supplementation with coffee as a nutritional ergogenic aid for trained female team-sport players during repeated sprint exercise.

## Figures and Tables

**Table 1:** Metabolic and physiological parameters assessed during a 12 × 4 s repeated sprint performance test with ingestion of placebo (PLA) or 3 mg·kg^−1^ of caffeine from coffee (LCOF) or 6 mg·kg^−1^ of caffeine from coffee (MCOF).

	Time Point	PLA	LCOF	MCOF
Lactate (mmol·L^−1^)	PreCof	1.8 ± 0.5	1.7 ± 0.5	1.8 ± 0.4
	PostRST	11.9 ± 1.3	13.8 ± 1.4 *	13.7 ± 1.6 *
Glucose (mmol·L^−1^)	PreCof	93.3 ± 8.0	101.9 ± 19.0	98.4 ± 10.4
	PostRST	119.1 ± 13.5	121.9 ± 15.1	117.0 ± 15.9
Heart rate (beat/min)	PreCof	67.0 ± 7.9	63.9 ± 5.5	64.4 ± 5.6
	MidRST	179.4 ± 5.5	180.3 ± 4.4	179.3 ± 5.3
	PostRST	180.4 ± 6.3	179.8 ± 4.6	180.4 ± 6.3
RPE (AU)	MidRST	15.1 ± 1.5	15.0 ± 1.5	15.2 ± 1.5
	PostRST	17.6 ± 1.6	17.6 ± 1.4	17.8 ± 1.7

*: Significantly different from PLA; PreCof: Prior to coffee ingestion; midRST: following 6 sprints; PostRST: after 12 sprints.

## Data Availability

The data presented in this study are available on request from the corresponding author. The data are not publicly available due to restrictions privacy.
